# Pressure dressings versus nonpressure dressings after hemorrhoidectomy: study protocol for a randomized controlled trial

**DOI:** 10.1186/s13063-021-05750-3

**Published:** 2021-11-13

**Authors:** Ping Xue, Jing Wu, Ping Zhu, Dan Wang, Mei Xu, Yi Zhang, Guanmao Lu, Quanyi Chen, Qin Zhang, Renjin Tang, Jinbo Fang

**Affiliations:** 1 West China School of Nursing, Sichuan University/ Department of Integrated Traditional Chinese and Western Medicine, West China Hospital, Sichuan University, Chengdu, Sichuan China; 2 Cheng Du Shang Jin Nan Fu Hospital, West China Hospital, Sichuan University, Chengdu, Sichuan China; 3 Department of Integrated Traditional Chinese and Western Medicine, West China Hospital, Sichuan University, Chengdu, Sichuan China

**Keywords:** Hemorrhoids, Hemorrhoidectomy, Urinary retention, Postoperative pain, Postoperative bleeding, Pressure dressing, Nonpressure dressing

## Abstract

**Background:**

Pressure dressings have been used after open hemorrhoidectomy to protect surgical wounds and manage postoperative bleeding for many years. However, pressure dressings may increase the incidence of postoperative complications, such as urinary retention, medical adhesive-related skin injury, and pain. A previous controlled trial included 67 patients who underwent Milligan-Morgan hemorrhoidectomy. The data indicated that the use of a nonpressure dressing after hemorrhoidectomy reduces the incidence of urinary retention and catheterization. However, the incidence of severe postoperative bleeding and other postoperative complications was not assessed. There is no consensus on whether it is necessary and beneficial to use a nonpressure dressing after hemorrhoidectomy. The results of this randomized clinical study will help answer this question.

**Methods:**

In this study, we plan to include 186 patients who have undergone modified Milligan-Morgan hemorrhoidectomy, which only sutured external hemorrhoids to reduce the risk of bleeding. The purpose is to determine whether the use of nonpressure dressings after open hemorrhoidectomy is inferior to the use of pressure dressings in terms of severe postoperative bleeding and postoperative complications. The primary endpoints of the trial are the incidence of urinary retention within 24 h after surgery and the incidence of severe postoperative bleeding 1 h after dressing removal, which requires revision surgery within 24 h after the surgery. The secondary endpoints of the study are the pain score, anal distension score, postoperative use of analgesics, and incidence of medical adhesive-related skin injury, all of which will be assessed before removing the dressings. The length of hospitalization in days and hospitalization expenses will be recorded. Safety will be assessed with consideration of all adverse and severe adverse events related to the study treatment.

**Discussion:**

The study received full ethics committee approval. The first patient was enrolled on 27 November 2020. The results of this trial will finally answer the question of whether a nonpressure dressing after open hemorrhoidectomy is necessary and beneficial.

**Trial registration:**

Chinese Clinical Trial Registry ChiCTR2000040283. Registered on 28 November 2020.

## Introduction

### Background and rationale {6a}

As people’s personal and work lifestyles change, the incidence of mixed hemorrhoids in China is increasing annually [1]. According to an epidemiological study, in 2016, the incidence of anorectal diseases among urban residents in China was 51.14%, and the incidence of mixed hemorrhoids was the highest among these diseases, accounting for 50.28% of all anorectal diseases [2]. In the USA, the incidence is approximately 4%, with the prevalence being highest between 45 and 65 years of age [3]. It is estimated that about 50% of Germans have symptomatic hemorrhoids, and some authors believe that up to 70% of people are affected by hemorrhoids [4]. Mixed hemorrhoids can occur at any age, and the symptoms include itching, pain, bleeding, swelling, prolapse of an anal tumor, foreign body sensation, etc. [5]. Although mixed hemorrhoids are not fatal or malignant, these symptoms can reduce a patient’s quality of life and have negative psychological and physical effects [6]. The treatment types include conservative treatment and surgical treatment. Mixed hemorrhoids with mild symptoms can be treated conservatively [7]. However, surgery is the initial treatment of choice in patients with symptomatic grade III–IV hemorrhoids [5, 6, 8]. Various surgical procedures have been used to treat symptomatic hemorrhoids in recent years, but (Milligan-Morgan) ligation is still considered the standard surgical procedure [9–13]. At the end of the operation, the wound is bandaged by a pressure dressing, which is the most traditional type of dressing that has been used clinically for many years [14]. The main purpose of pressure bandaging is to stop bleeding, but it may increase the incidence of postoperative complications, such as urinary retention, pain, anal swelling, and medical adhesive-related skin damage [12, 15–18]. Besides, clinical observation shows that with medical advances and the application of hemostatic drugs, the incidence of active bleeding within 24 h after modified Milligan-Morgan hemorrhoids surgery is very low. In addition, the necessity of pressure dressing after hemorrhoidectomy has received little scientific attention. A previous controlled trial included 67 patients who underwent Milligan-Morgan hemorrhoidectomy [19]. The data indicated that the use of a nonpressure dressing after hemorrhoidectomy reduces the incidence of urinary retention and catheterization. However, the incidence of severe postoperative bleeding and other postoperative complications was not assessed. There is no consensus on whether it is necessary and beneficial to use a nonpressure dressing after hemorrhoidectomy. The results of this randomized clinical study will preliminarily help answer this question.

### Objectives {7}

#### Primary objectives

1. Incidence of retention of urine within 24 h after operation

#### Secondary objectives

To assess the impact of pressure dressings versus nonpressure dressings surveillance on:

1. Pain score within 25 h after operation (6H\18H\25H);
2. The change in hemostatic effect scores within 25 h after operation;
3. The incidence of medically adhesive skin lesions;
4. The anal edema score within 25 h after operation (6H\18H\25H);
5. The anal dilatation score within 25 h after operation (6H\18H\25H);
6. Dose of analgesics within 24 h after operation;
7. Hospitalization costs; and
8. Length of stay.

To assess safety, the incidence of adverse events (AEs) and severe adverse events (SAEs) between the two study groups will be compared to illustrate the potential risk to patients with nonpressure dressings after hemorrhoidectomy.

### Trial design {8,9}

**Fig. 1:**
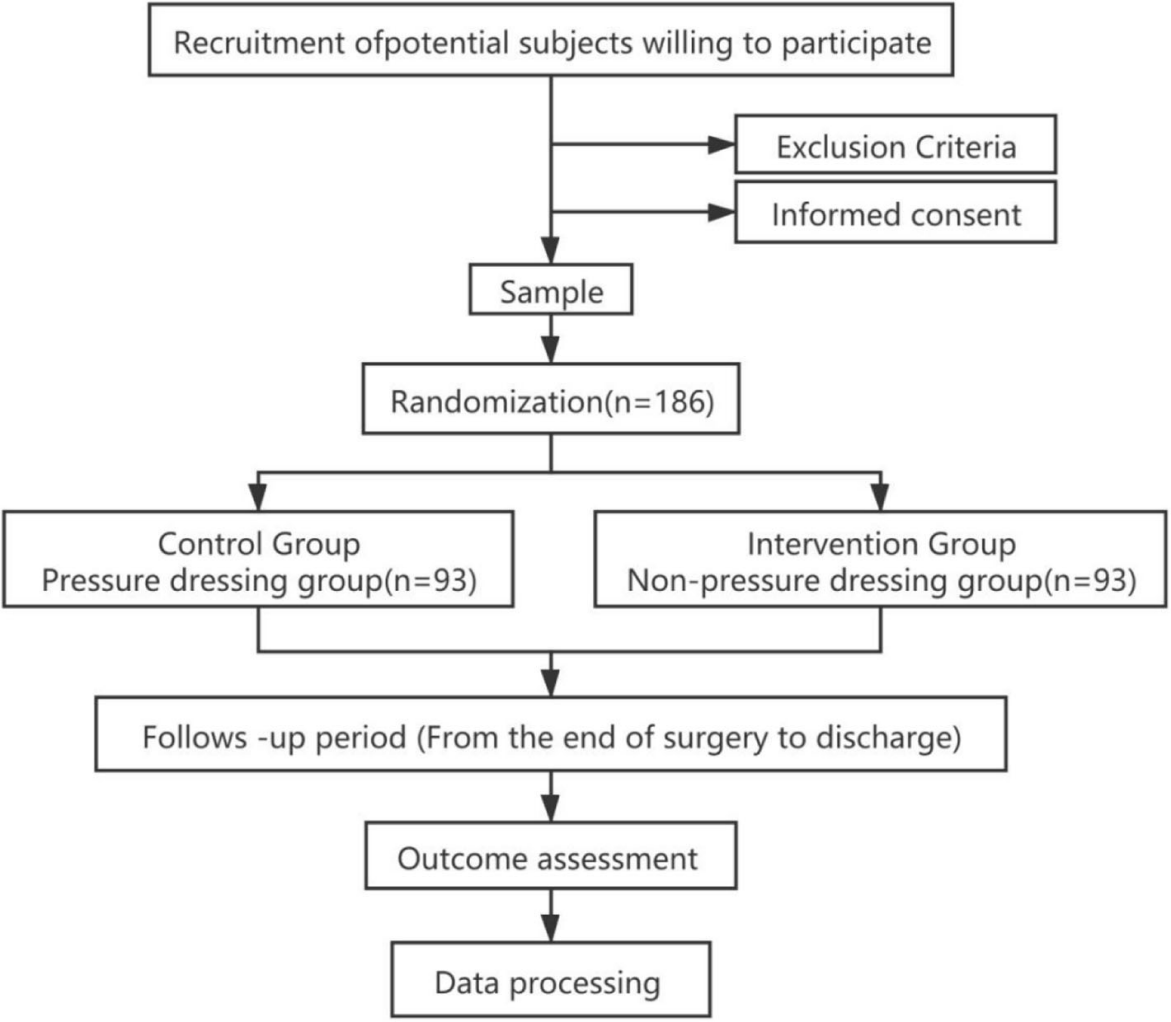
A flow chart of the study stages

This is a single-blind, randomized controlled study that will be conducted in the Department of Integrated Traditional Chinese and Western Medicine at West China Hospital in Sichuan University, China (Fig. 1). Eligible participants who meet the clinical criteria for mixed hemorrhoids of the Professional Committee of Colorectal Diseases, Chinese Society of Integrated Chinese and Western Medicine will be enrolled. The trial was approved by the Ethics Committee on Biomedical Research at West China Hospital of Sichuan University (approval code: NO. 2020-549). It was also registered in the Chinese Clinical Trial Registry (registration ID: ChiCTR2000040283).

**Fig. 2:**
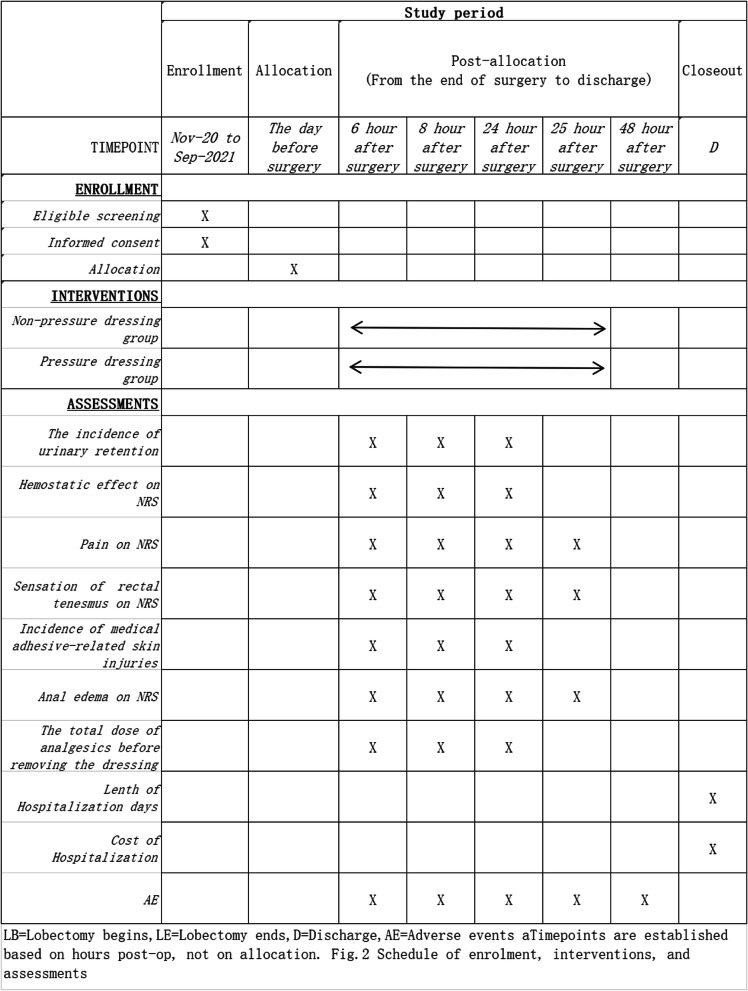
Timeline of the enrollment process, interventions, and assessments. Note: D, discharge; AE, adverse events; NRS, numerical rating scale

A total of 186 participants with mixed hemorrhoids will be randomly assigned to two groups at a 1:1 ratio. After randomization, the participants in the two groups will undergo surgical treatment with modified Milligan-Morgan hemorrhoidectomy, which only sutured external hemorrhoids to reduce the risk of bleeding under general anesthesia. The incision for external hemorrhoids is fully sutured to stop bleeding. There is no active bleeding after surgery. The experimental group and control group will be treated with nonpressure dressings and pressure dressings, respectively. The timeline for the enrollment process, intervention, and follow-ups is summarized in a SPIRIT figure (Fig. 2). {13}

### Eligibility criteria {10}

#### Inclusion criteria

The inclusion criteria are as follows:

1. Grade III–IV mixed hemorrhoids;
2. Aged 18~65 years old;
3. Having the willingness to cooperate during the study;
4. Having provided informed consent; and
5. The absence of a history of surgery for anorectal diseases. Note: Only the patients who meet the above 5 items will be included in the study.

#### Exclusion criteria

The exclusion criteria are as follows:

1. Patients with degree IV circular mixed hemorrhoids;
2. Women who are breastfeeding, pregnant, or menstruating;
3. Patients with tuberculosis, diabetes, or cardiovascular, cerebrovascular, liver, kidney, or hematopoietic system disorders;
4. Patients with anorectal inflammation, perianal abscess, tumor, ulcerative colitis, Crohn’s disease, perianal skin disease, or related conditions;
5. Fecal incontinence patients with Wexner score greater than 0 [20]; and
6. Patients in whom the curative effect cannot be determined or who have incomplete data.

### Sample size {14}

The results of this study will be compared with the two-sided, two-sample chi-square test. The sample size should provide 80% statistical power at a significance level of 0.05, and a sample size ratio of 1:1. In the preliminary trial, the incidence of urinary retention was the main outcome measure and was 44.7% in the nonpressure dressing group and 24.7% in the pressure dressing group. Considering an anticipated dropout rate of 10%, we plan to include a total of 186 participants, with 93 participants per group.

### Study setting {9}

This trial will take place at the department of integrated traditional Chinese and Western medicine, West China Hospital, Sichuan University, China.

### Recruitment {15}

Researchers will recruit participants from the inpatient wards of Integrated Traditional Chinese and Western Medicine Departments. Patients eligible for the trial must meet all the inclusion criteria and exclusion criteria before enrollment. To recruit enough participants, all doctors in these departments will be informed of the trial and will be asked to contact the research assistant if they encounter potentially eligible patients. Potential participants will be screened, receive information on the trial, and then, be asked to sign informed consent forms. Patients who meet the selection criteria will undergo baseline assessments, and the eligible patients will be asked by medical staff to complete a general information form including their name, sex, age, medical history, etc.

## Randomization and blinding

Randomization will be performed by an independent statistician with no clinical involvement in this trial by use of a random number generator program in Excel software (Kingsoft Office Software Co. Ltd., China). A simple randomization method and table of random numbers will be used. If the selected number is even, the patient is allocated to the nonpressure dressings group, and if it is odd, the patient is allocated to the pressure dressings group in a 1:1 ratio. Only one data administrator can access the random number table on the computer. An independent staff member who will not take part in the performance of this study will seal the random numbers in opaque envelopes. Then, the primary investigator will save these envelopes and open one of them when a participant is enrolled.

The participants will be enrolled by nursing staff and research assistants in the order of admission time. And they will be grouped by the data administrator using the table of random numbers. The two groups will use different types of dressings, and the operators will place the dressings. The patients can feel whether the wound is pressurized, so they cannot be blinded. However, the operators will remove the dressing before the observer’s follow-up. The observers and statisticians will be blinded to the group assignments.

### Relevant concomitant care permitted or prohibited during the trial {11d}

Both groups of patients will receive conventional treatment and care during the perioperative period. These treatments will include fasting and drinking before surgery, the use of glycerine enema and fluid replacement, the postoperative use of analgesics, intravenous analgesia pumps, and hemostatic drugs, if necessary. Pain therapy and prophylaxis will include muscle relaxants, local anesthetics, anti-inflammatory drugs, opioids, nonsteroidal medications, and other medications. During the postoperative period, the patient may receive intravenous or oral pain medication as needed. All previous and concomitant medications will be administered as per clinical routine and according to the patient’s needs and demands. All information regarding the use of analgesic drugs will be recorded. Both groups of patients will use the same dose of hemocoagulase for preventive hemostasis. Prior to the commencement of the study, all investigators will receive standardized training on trial content, treatment strategies, evaluation, and quality control. Interventions will be carried out in accordance with good clinical practice guidelines.

### Intervention description {11a}

#### Nonpressure dressing group

In the nonpressure dressing group, gauze will be placed on the operative wounds of the patient after complete hemostasis is achieved, and then, the dressing will be placed on the gauze with foam adhesive dressing to avoid the perineum area. The purpose of the small layer of gauze is to prevent the foam adhesive dressing from affecting the wound and reduce bias.

#### Pressure dressing group

In the pressure dressing group, an operator will compress the wound with three layers of gauze (AM 9 cm × 11 cm × 12 cm) in a “tower” shape after achieving complete hemostasis. First, one layer of gauze will be folded twice over the surgical wound. Second, it will be covered directly with two layers of gauze and two pieces of silk tape (45 cm × 2.5 cm, 3M China Co., Ltd.) in a “Y” shape to press the gauze firmly. The two pieces of silk tape will be fixed on both sides of the groin toward the middle of the back with moderate strength, avoiding the perineum during application.

### Ancillary and post-trial care {30}

All participants will return to usual care after dressing removal at postoperative 24 h. As part of our safety protocol, we will monitor patients’ surgical wound pain, micturition, skin damage, and active bleeding. Any participants with the above abnormal situations will be contacted by the site coordinator who will inform the doctor for timely treatment.

#### Discontinuations

If participants are unable or unwilling to complete the treatment, or the doctor’s assessment suggests that it is inappropriate to continue the study intervention due to a severe adverse event, we will consider them to be treatment dropouts.

### Outcome measures {12}

#### Primary outcome measures

1. There will be two primary outcomes. The incidence of urinary retention within 24 h will be one of these; typically, patients cannot urinate by themselves after using a hot compress or drug assistance and finally undergo retention catheterization.

#### Secondary outcome measures

1. Postoperative pain will be measured at rest with routine care at 6, 18, and 25 h by using a visual analog scale (VAS) after the surgical procedure. The scale will range from 0 to 10 points, with higher scores indicating more severe pain [21]: 0 = no pain, 1~3 = slight pain that can be tolerated, 4~6 = pain that can be tolerated but affects sleep, and 7~10 = unbearable pain that affects not only appetite but also sleep.
2. The change in hemostatic effect scores within 25 h will be the other primary outcome. The effect of hemostasis will be graded using a 3-point ordinal scale: 0 = the dressing was not soaked before removal, 1 = there was no bleeding after changing the dressing due to excessive blood oozing, and 2 = postoperative bleeding requiring surgical revision occurred.
3. The change in mean rectal tenesmus sensation scores within 25 h will be graded using a 3-point ordinal scale: 0 = no abnormal sensation; 1 = mild anal discomfort, occasionally abnormal sensation; 2 = strong foreign-body sensation; and 3 = strong foreign body sensation that is not easily relieved after rest or treatment.
4. The incidence of medical adhesive-related skin injuries will be measured. The skin condition will be assessed by a senior wound specialist nurse. If there is no skin damage, “no” will be recorded. When any of the following situations occur, “yes” will be recorded: (1) exfoliation, (2) tension blisters, (3) skin lacerations, (4) contact dermatitis, and (5) allergic dermatitis. In the statistical analysis, the incidence of medical adhesive-related skin damage will be calculated by a statistician.
5. The change in the mean anal edema score when the dressing is removed 24 h after surgery will be graded using a 4-point ordinal scale: 0 = no anal edema, 1 = mild edema (< 1/4 anal area ), 2 = moderate edema (1/4 to 1/2 anal area ), and 3 = severe edema (> 1/2 anal area ).
6. The total dose of analgesics before removing the dressing will be recorded.
7. The length of hospitalization will be recorded in days.
8. Hospitalization expenses will be recorded.

## Study organization

### Data collection and management {19}

The researchers will independently record demographic and clinical data for all patients. Preoperative data will be collected within 1 day after recruitment. Postoperative data will be collected within 2 days after surgery. The organizational structure of the trial is as follows. The steering committee will have full oversight over the design of the trial. The investigators of the Data Management Safety Committee (DMSC) will supervise and confirm that the case report form (CRF) is correctly completed and that the data are consistent with the original data. If there are any errors or omissions, the investigator will immediately correct them. We do not intend to collect personal information about potential or registered participants other than that typically collected during hospitalization. For confidentiality, the electronic health information will be encrypted in accordance with the hospital protocol. After the trial, personally identifiable information will be omitted and placed in a separate database for data analysis.

### Statistical analysis

The validity analysis of the study will be mainly based on intention-to-treat (ITT, all randomized cases) and per-protocol analyses (PP, cases that comply with the trial protocol, good compliance, and completed CRF). First, the Shapiro-Wilk test will be used to test whether the quantitative data follow a normal distribution. If the data are normally distributed, the mean value and standard deviation will be used to describe the quantitative data, and a *t* test will be used for analysis. Nonnormally distributed data will be expressed as the median and quaternary and compared using the Mann-Whitney *U* test. The qualitative data will be expressed as the frequency and percentage; non-grade data will be compared by the chi-square test, and grade data will be compared by the Mann-Whitney *U* test. Randomization will be performed 24 h prior to the surgical intervention. Data will not be collected and analyzed for patients not undergoing surgical intervention. If the scheduled surgery is not performed, the study participant will be excluded from the clinical trial. For every patient who is excluded from the trial after randomization and before the start of the observation period (postoperative wound with or without compression), another study participant will be included to achieve the number of cases needed. The randomization sequence will continue. The next patient will not necessarily be assigned to the same group as the excluded patient. This research will continue until the required number of evaluable patients is reached. Due to complications, a very small number of patients may not be able to comply with the randomly assigned treatment. The study will not exclude these patients. All statistical tests will be two-sided, and *P* < 0.05 will be considered statistically significant. If baseline factors are inconsistent between the two groups, subgroup analysis and multivariate analysis will be considered.

In the safety analysis, Fisher’s exact test will be used to compare the incidence of AEs and SAEs by category (severity) between the two groups.

### Adverse events (AEs) {22}

In this study, AEs are defined as any treatment-related medical events, including any treatment-related adverse and unexpected signs, symptoms, or diseases. The AEs known to follow nonpressure dressing and pressure dressing treatment include severe postoperative hemorrhage. At each visit, the participants will report AEs and be examined by a physician. AEs will be evaluated and reported to the principal investigator according to Common Terminology Criteria for Adverse Events (v4.03) [22].

### Auditing {23}

The ethics committee will conduct local monitoring of trial quality after the first patients have been enrolled. The Trial Management Team will meet every 3 months to check the implementation of the study, including the recruitment rate, data quality, and adverse event reporting.

### Plans for communicating important protocol amendments to relevant parties {25}

The protocol, statistical analysis plan, data safety management plan, informed consent forms, and recruitment materials have been reviewed and approved by the Ethics Committee on Biomedical Research at West China Hospital of Sichuan University. Any subsequent modifications will be submitted for review, and annual safety and progress reports will be presented. In addition, online trial registries will be updated accordingly. The data will be accessible through the research center upon reasonable request.

### Dissemination plans {31a}

The outcomes will be disseminated through peer-reviewed publications, a master’s thesis, community groups, or conference presentations. The data will be published on the website of the Clinical Research Management Department of West China Hospital of Sichuan University. Anyone who needs data can contact the author.

## Discussion

Pressure dressings are commonly used and have been considered a clinical standard for wounds after mixed hemorrhoid operations in China. However, these dressings can lead to some postoperative complications, and there are few reports on improvements in wound dressing methods after hemorrhoid surgery. This study will be carried out to determine whether nonpressure bandaging can be used routinely after open hemorrhoidectomy and whether it may be considered in specific circumstances.

During the operation, the patients will be treated with general anesthesia, and the method of applying a pressure dressing is complicated, so at least two medical staff will perform wound bandaging; nonpressure dressings need only a self-adhesive foam dressing, and only one medical staff member will be needed to perform wound dressing, and the process takes less time. The nonpressure dressing method can not only reduce the workload of medical staff but also save time.

Methods to minimize bias were implemented whenever possible. Randomization was performed 24 h before surgery. Randomization ensures a statistically consistent baseline between the experimental and control groups. However, one limitation of this study is that the researchers and patients cannot be blinded to the group assignments. The blinding of patients is an important way to prevent bias, but unfortunately, this step cannot be carried out in this study. However, the observers can be blinded. Before conducting any trial-related procedures, the researcher will obtain a written informed consent from each potential research participant. Randomization will be performed with a random number table generated in Excel, and then, the patients will be assigned a group according to the order of admission.

The research plan is consistent with clinical practice to the greatest extent possible to ensure complete and trouble-free data collection. Observation indicators such as pain and anal swelling may have affected the evaluation at certain time points while the patient is sleeping. Missing data are expected in these cases, but they should be minimized. The research leader will bear full legal responsibility for the entire study and publish the data on the website of the Clinical Research Management Department of West China Hospital of Sichuan University. Anyone who needs data can contact the author.

## Trial status

Participant recruitment is still being undertaken. Enrollment started in November 2020, and the trial is expected to be completed by December 2021.

## Abbreviations

AE: Adverse event
CI: Confidence interval
CRF: Case report forms
ITT: Intention to treat
NRS: Numerical rating scale
D: Discharge
PP: Per protocol
RCT: Randomized controlled trial
SAE: Severe adverse event
SD: Standard deviation
